# Tracking Taphonomic Regimes Using Chemical and Mechanical Damage of Pollen and Spores: An Example from the Triassic–Jurassic Mass Extinction

**DOI:** 10.1371/journal.pone.0049153

**Published:** 2012-11-07

**Authors:** Luke Mander, Cassandra J. Wesseln, Jennifer C. McElwain, Surangi W. Punyasena

**Affiliations:** 1 Department of Plant Biology, University of Illinois, Urbana, Illinois, United States of America; 2 School of Biology and Environmental Science, University College Dublin, Belfield, Dublin, Ireland; Ludwig-Maximilians-Universität München, Germany

## Abstract

The interpretation of biotic changes in the geological past relies on the assumption that samples from different time intervals represent an equivalent suite of natural sampling conditions. As a result, detailed investigations of taphonomic regimes during intervals of major biotic upheaval, such as mass extinctions, are crucial. In this paper, we have used variations in the frequency of chemical and mechanical sporomorph (pollen and spore) damage as a guide to taphonomic regimes across the Triassic–Jurassic mass extinction (Tr-J; ∼201.3 Ma) at a boundary section at Astartekløft, East Greenland. We find that the frequency of sporomorph damage is extremely variable in samples from this locality. This likely reflects a combination of taxon-specific susceptibility to damage and the mixing of sporomorphs from a mosaic of environments and taphonomic regimes. The stratigraphic interval containing evidence of plant extinction and compositional change in the source vegetation at Astartekløft is not marked by a consistent rise or fall in the frequency of sporomorph damage. This indicates that natural taphonomic regimes did not shift radically during this critical interval. We find no evidence of a consistent relationship between the taxonomic richness of sporomorph assemblages and the frequency of damage among sporomorphs at Astartekløft. This indicates that previously reported patterns of sporomorph richness across the Tr-J at this locality are likely to be robust. Taken together, our results suggest that the patterns of vegetation change at Astartekløft represent a real biological response to environmental change at the Tr-J.

## Introduction

During the Triassic–Jurassic transition (Tr-J; ∼201.3 Ma [1]) the Earth’s biota underwent a reorganisation that culminated in one of the ‘Big-5′ mass extinction events of the Phanerozoic [2]. Extinction at the Tr-J was likely related to the environmental effects of volcanism in the Central Atlantic Magmatic Province (CAMP) [3], [4], which are thought to have included rising atmospheric CO_2_ levels and global warming [5]–[8], release of volcanic pollutants such as SO_2_ [9], and the emission of thermogenic methane [10]. Plant fossils from a Tr-J boundary section at Astartekløft in East Greenland have provided information on the regional response of Earth’s vegetation to environmental change at the Tr-J. Macrofossils (mostly leaves) preserve a ∼17% genus-level extinction and an abrupt decline in terrestrial plant diversity [11], [12]. Insect-pollinated plants were at the greatest risk of extinction [11] and the pace of plant diversity loss in East Greenland was rapid [12]. Sporomorph (pollen and spore) assemblages from this region record a 10–12% decline in taxonomic diversity in the Tr-J boundary interval, and there is evidence of compositional change driven by emigration and/or extirpation of plants [13].

**Figure 1:**
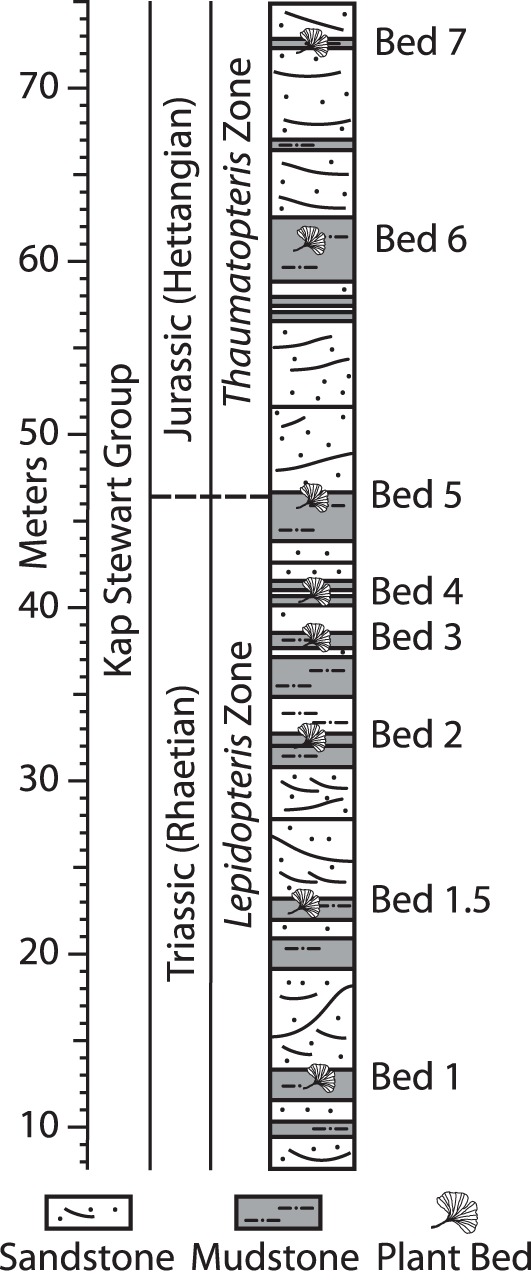
Schematic sedimentary log of the Astartekløft section (adapted from [**11**], [**42**], and modified from [**13**], [43], [**44**]). Stratigraphic position of plant beds from [11]. Plant beds represented by schematic *Ginkgo* leaves. Position of Triassic/Jurassic boundary approximated by the first appearance of *Cerebropollenites thiergartii* following [45], [46] (see [13]). *Lepidopteris* and *Thaumatopteris* macrofossil leaf zones from [16] (see also [11]).

However, the interpretation of changes in taxonomic diversity or composition over time relies on the assumption that the information from each sample is derived from an equivalent suite of natural sampling conditions [14]. As a result, detailed investigations of taphonomic regimes through an interval of presumed biotic change are crucial [14], [15]. Previous work on taphonomic regimes at Astartekløft has focused on the environments of deposition. Plant fossils at Astartekløft are restricted to a series of muddy and silty horizons referred to as “plant beds” [11], [16] (Figure 1). Plant beds 1–5 all represent deposition by floodwaters into overbank environments (crevasse splays) [11] and are therefore “somewhat isotaphonomic by default” ([14], p. 131). For this reason, vegetation changes that have been recorded within plant beds 1–5, such as the 10–12% decline in sporomorph diversity and compositional change at the Tr-J boundary in bed 5, are thought to be largely free from taphonomic control [11], [13]. In contrast, plant bed 6 represents a poorly developed coal swamp and plant bed 7 represents a shallow pool developed in a semi-abandoned channel [11], and this represents a first-order change in sampling conditions at this locality [11]. As a result, differences in plant diversity and vegetation composition between plant beds 1–5 and plant beds 6–7 have been interpreted with caution [11], [13].

This assessment of taphonomic regimes at Astartekløft is based on outcrop observations of lithology and sedimentology [11]. Consequently, the nature and distribution of processes such as chemical corrosion and mechanical damage of organisms, which can be important components of the taphonomic regime in a rock succession [14], is currently unclear. These processes are reflected in dispersed sporomorph assemblages [17], [18], and sporomorphs encountered in palynological preparations may be chemically corroded by oxidation or microbial attack [19], or mechanically damaged by tearing, compression or folding as a result of repeated exposure to wet and dry cycles [20]. Reworking can also lead to appreciable damage, although re-worked sporomorphs are not always more poorly preserved than non-reworked sporomorphs [18].

In this paper we use variations in the frequency of chemical and mechanical sporomorph damage as a guide to taphonomic regimes across the Tr-J at Astartekløft. We aim to assess the degree to which each plant bed at this locality represents an equivalent suite of natural sampling conditions by providing the following: (1) baseline data on the frequency of chemical and mechanical sporomorph damage across the Tr-J at Astartekløft; (2) an examination of the relationship between the taxonomic richness of sporomorph assemblages and the frequency of sporomorph damage at Astartekløft.

## Materials and Methods

### Ethics Statement

A permit was obtained from the Geological Survey of Denmark and Greenland to collect rock samples from Greenland (GEUS reference number 512–220). The land accessed is not privately owned or protected. No living material was sampled. No protected species were sampled.

### Geological Setting and Palynology

Astartekløft is a cliff section in Jameson Land, East Greenland that has yielded diverse and exceptionally well-preserved plant macrofossils [11], [16] and sporomorphs [13], [21], [22]. Eighteen samples from plant beds 3–7 were analysed in this study (Figure 1; Table 1). These samples were selected to encompass a wide range of within-sample richness values reported in [13] (14.57 to 38.51 taxa; see Table 1). Samples from plant bed 6 consist of coaly mudstone, and samples from all other plant beds consist of dark grey mudstones and siltstones. Between 15 and 20 g of each sample was washed and crushed and dried for 24 hours at 60°C. Each sample was treated twice alternately with cold HCl (30%) to remove carbonates, and with cold HF (38%) to remove silicates. The residue from each sample was washed with water until pH neutral, then sieved with 250 µm and 15 µm polypropylene mesh. Finally, organic and inorganic residues were separated using ZnCl_2_. No oxidation techniques were used during the preparation of sporomorphs. Slide preparations were made in glycerine jelly.

**Table 1:** Details of the 18 samples investigated in this study.

				Taxa Present			
Environment of Deposition	Plant Bed	Sample	Within-Sample Richness	*Deltoidospora*	*Baculatisporites*	*Uvaesporites*	Random
Abandoned Channel	7	7_7269	38.51	x	✓	x	✓
		7_7259	21.92	x	✓	x	✓
		7_7239	32.09	x	✓	x	✓
Coal Swamp	6	6_6106	17.14	✓	x	x	✓
		6_6086	24.46	✓	x	x	✓
		6_6076	22.41	✓	x	x	✓
Overbank	5	5_4718	31.40	x	✓	✓	✓
		5_4678	18.97	x	✓	✓	✓
		5_4668	25.27	x	✓	✓	✓
		5_4658	23.99	x	✓	✓	✓
		5_4648	23.33	x	✓	✓	✓
		5_4638	14.57	x	✓	x	✓
Overbank	4	4_4107	27.87	✓	x	x	✓
		4_4077	23.04	✓	x	x	✓
		4_4067	30.10	✓	x	x	✓
Overbank	3	3_3771	23.54	x	✓	✓	✓
		3_3761	31.38	x	✓	✓	✓
		3_3715	19.40	✓	x	x	✓

Environments of deposition from [11], [13]. Sample numbers as follows: [plant bed number]_[stratigraphic height in centimetres]. Stratigraphic height from the sedimentary log of [42] (see also [11]) (Figure 1). Within-sample richness from [13].

### Assessing the Preservation of Sporomorphs from Astartekløft

From each sample, randomly selected sporomorph specimens and (where present) specimens of three spore genera *Deltoidospora*, *Baculatisporites* and *Uvaesporites* were assessed for the presence or absence of five damage types. These damage types were: thinning; corrosion; breakage; pinching; folding. Sporomorphs that were unaffected by any of these damage types were scored as “perfect”. Sporomorphs were often scored for the presence of multiple damage types. These damage states were selected from schemes developed for the study of pollen preservation in Quaternary sediments [23]–[26] to reflect the nature and range of sporomorph damage observed in our samples. More than 55 sporomorph specimens of each category were examined per sample (Table 2, 3), although only 36 specimens of *Uvaesporites* were analysed in sample 5_4678 owing to the low abundance of this taxon in this sample (Table 3). Each specimen was examined under transmitted light at 400× magnification using an ECPlan-Neofluar 40× objective (Numerical Aperture 0.75).

**Table 2:** Damage State: Random Sporomorphs.

		Perfect		Thinned		Corroded		Broken		Pinched		Folded	
Sample	*n* Total	*n*	%	*n*	%	*n*	%	*n*	%	*n*	%	*n*	%
7_7269	60	0	0.00	34	56.67	24	40.00	18	30.00	33	55.00	19	31.67
7_7259	60	0	0.00	24	40.00	16	26.67	25	41.67	38	63.33	17	28.33
7_7239	58	1	1.72	36	62.07	21	36.21	16	27.59	43	74.14	11	18.97
6_6106	58	1	1.72	11	18.97	0	0.00	19	32.76	35	60.34	12	20.69
6_6086	60	1	1.67	1	1.67	4	6.67	12	20.00	45	75.00	12	20.00
6_6076	59	2	3.39	5	8.47	1	1.69	15	25.42	31	52.54	17	28.81
5_4718	59	1	1.69	40	67.80	24	40.68	21	35.59	29	49.15	13	22.03
5_4678	60	0	0.00	36	60.00	26	43.33	28	46.67	28	46.67	24	40.00
5_4668	60	0	0.00	37	61.67	28	46.67	24	40.00	32	53.33	23	38.33
5_4658	60	0	0.00	36	60.00	19	31.67	27	45.00	26	43.33	25	41.67
5_4648	60	1	1.67	31	51.67	16	26.67	19	31.67	24	40.00	27	45.00
5_4638	60	2	3.33	15	25.00	4	6.67	31	51.67	17	28.33	28	46.67
4_4107	58	1	1.72	21	36.21	8	13.79	13	22.41	36	62.07	17	29.31
4_4077	60	0	0.00	26	43.33	9	15.00	25	41.67	19	31.67	28	46.67
4_4067	61	1	1.64	24	39.34	10	16.39	21	34.43	21	34.43	22	36.07
3_3771	62	0	0.00	34	54.84	8	12.90	29	46.77	21	33.87	25	40.32
3_3761	61	0	0.00	24	39.34	7	11.48	28	45.90	24	39.34	23	37.70
3_3715	61	1	1.64	34	55.74	25	40.98	25	40.98	18	29.51	26	42.62

Data matrix of the frequency of each damage type in random sporomorphs from each of the 18 samples investigated here. Details of sample numbering as for Table 1. *n* Total represents the total number of individual sporomorphs examined for the presence of each damage type in each sample. *n* represents the total number of sporomorphs in each sample that were scored as perfect or damaged (expressed as a percentage in the column headed “%”).

**Figure 2:**
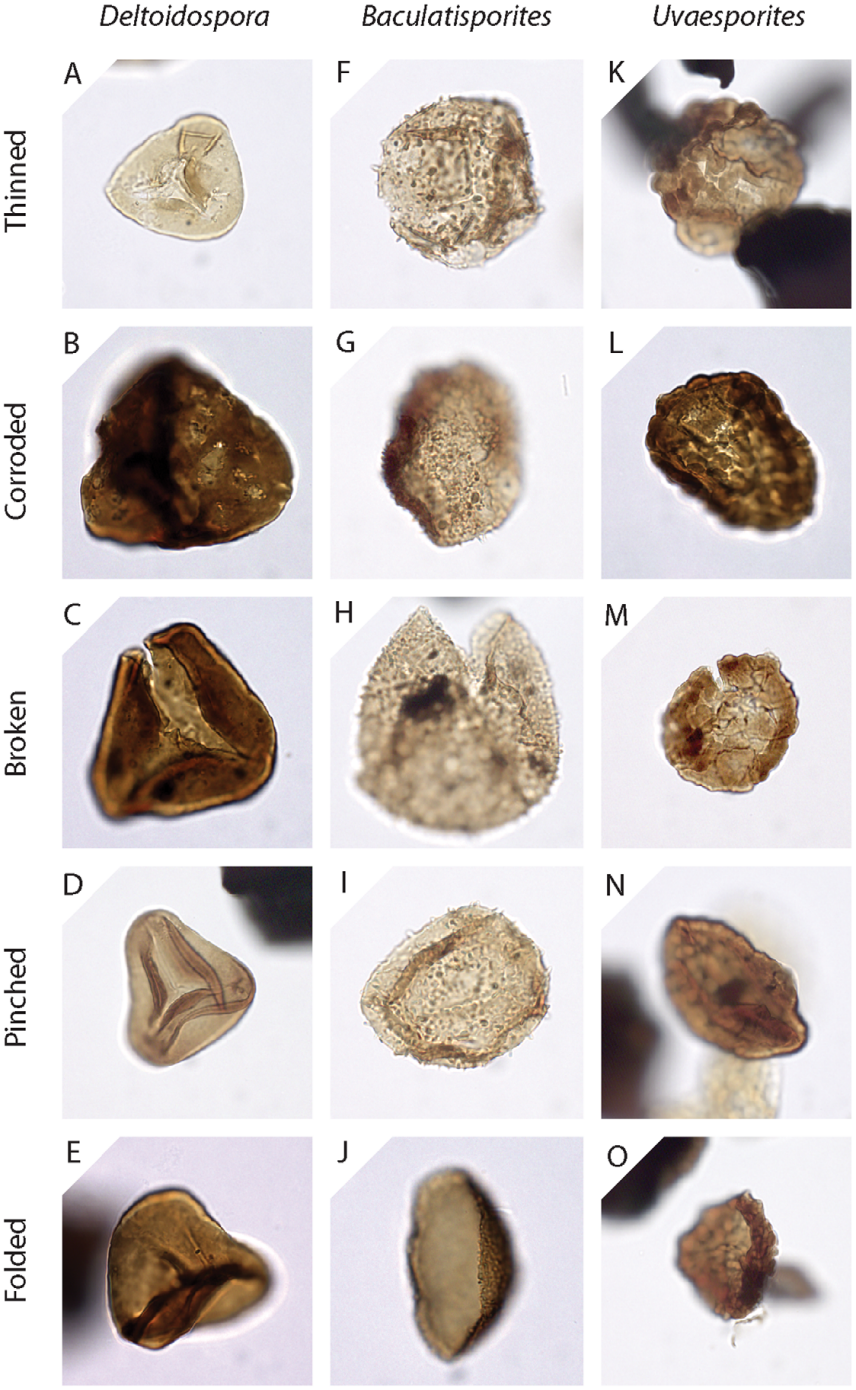
Representative images of the 5 damage types expressed in *Deltoidospora* (4A–E), *Baculatisporites* (4F–J) and *Uvaesporites* (4K–O). No perfect grains of *Deltoidospora* or *Baculatisporites* were found during the course of this study, perfect grains of *Uvaesporites* not shown.

The preservation state of each specimen was assessed as follows. If the sporomorph exine was distinctly more transparent than usual for the sporomorph taxon, the presence of the thinned damage type was recorded. If the sporomorph exine was marked by small depressions or holes that were not primary morphological features of the sporomorph, the presence of the corroded damage type was recorded. Sporomorphs with a broken exine were scored for the presence of the broken damage type. If folding disrupted the outline of the sporomorph, the sporomorph was marked for the presence of the folded damage type. However, if folding was present but did not disrupt the outline of the sporomorph, then the specimen was marked for the presence of the pinched damage type. Holotype images of each damage state are shown in Figure 2. Each holotype image depicts an ideal example of a particular damage state, but multiple damage types affected many sporomorphs.

## Results

### The Frequency of Sporomorph Damage at Astartekløft

**Figure 3:**
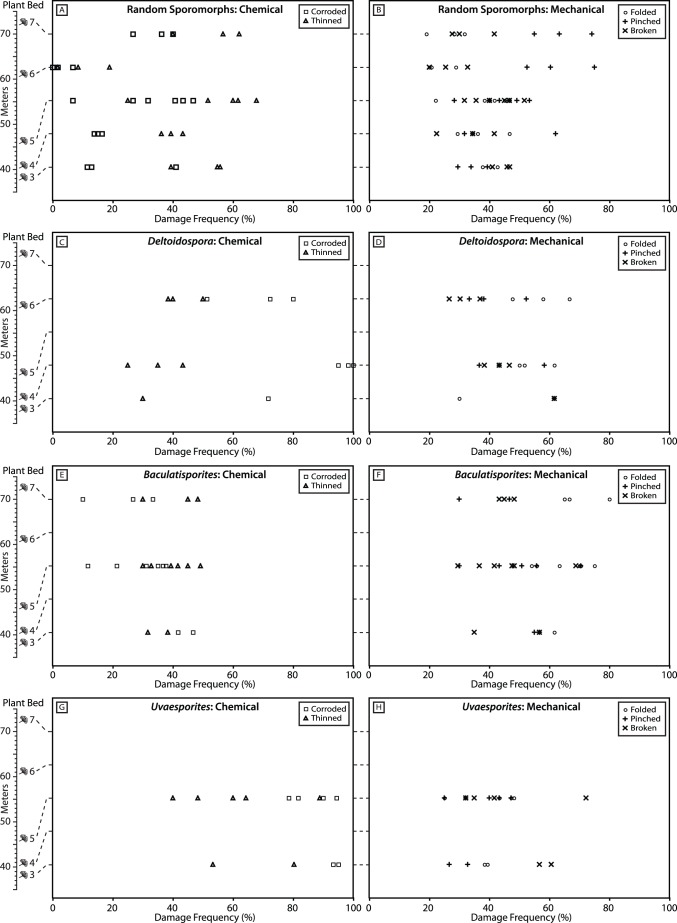
Dotplots showing the frequency of each damage type in each of the plant beds investigated here. Stratigraphic position of plant beds from [11]. Plant beds represented by schematic *Ginkgo* leaves (see Figure 1). Figures 4A, C, E and G show the frequency of chemical damage (thinning and corrosion) and Figures 4B, D, F and H show the frequency of mechanical damage (folding, pinching and breaking). Perfect grains not shown.

The frequency of each sporomorph damage type is extremely variable in samples from the plant beds at Astartekløft. Among randomly selected sporomorphs, the frequency of chemical damage ranges from 0 to 68% (Figure 3A, Table 2), and the frequency of mechanical damage ranges from 19 to 75% (Figure 3B, Table 2). This variability is also evident in the frequency of each damage type in the three spore genera *Deltoidospora*, *Baculatisporites* and *Uvaesporites* (Figure 3, Table 3). Examples of notably variable damage frequency include corrosion in randomly selected sporomorphs from plant bed 5 (Figure 3A), corrosion in specimens of *Deltoidospora* from plant bed 6 (Figure 3C), and breakage in specimens of *Baculatisporites* from plant bed 5 (Figure 3F). Occasionally, the frequency of sporomorph damage in a set of samples is clustered rather than variable. Examples of this include corrosion among randomly selected sporomorphs and specimens of *Deltoidospora* from plant bed 4 (Figs. 3A, C), and folding among randomly selected sporomorphs from plant bed 3 (Figure 3B). The frequency of corrosion is consistently high in *Deltoidospora* and *Uvaesporites*, but is low in *Baculatisporites* (Figure 3).

**Table 3:** Damage State: *Deltoidospora.*

		Perfect		Thinned		Corroded		Broken		Pinched		Folded	
Sample	*n* Total	*n*	%	*n*	%	*n*	%	*n*	%	*n*	%	*n*	%
6_6106	76	*0*	0.00	*38*	50.00	*39*	51.32	*23*	30.26	*29*	38.16	*44*	57.89
6_6086	65	*0*	0.00	*25*	38.46	*47*	72.31	*24*	36.92	*34*	52.31	*31*	47.69
6_6076	60	*0*	0.00	*24*	40.00	*48*	80.00	*16*	26.67	*20*	33.33	*40*	66.67
4_4107	60	*0*	0.00	*21*	35.00	*57*	95.00	*26*	43.33	*22*	36.67	*30*	50.00
4_4077	60	*0*	0.00	*26*	43.33	*60*	100.00	*28*	46.67	*26*	43.33	*37*	61.67
4_4067	60	*0*	0.00	*15*	25.00	*59*	98.33	*23*	38.33	*35*	58.33	*31*	51.67
3_3715	60	*0*	0.00	*18*	30.00	*43*	71.67	*37*	61.67	*37*	61.67	*18*	30.00
		**Damage State: ** ***Baculatisporites***											
		**Perfect**		**Thinned**		**Corroded**		**Broken**		**Pinched**		**Folded**	
**Sample**	***n*** ** Total**	***n***	**%**	***n***	**%**	***n***	**%**	***n***	**%**	***n***	**%**	***n***	**%**
7_7269	60	*0*	0.00	*18*	30.00	*6*	10.00	*29*	48.33	*28*	46.67	*40*	66.67
7_7259	60	*0*	0.00	*29*	48.33	*16*	26.67	*26*	43.33	*18*	30.00	*48*	80.00
7_7239	60	*0*	0.00	*27*	45.00	*20*	33.33	*27*	45.00	*28*	46.67	*39*	65.00
5_4718	61	*0*	0.00	*24*	39.34	*13*	21.31	*29*	47.54	*34*	55.74	*34*	55.74
5_4678	61	*0*	0.00	*30*	49.18	*23*	37.70	*42*	68.85	*43*	70.49	*33*	54.10
5_4668	60	*0*	0.00	*27*	45.00	*22*	36.67	*29*	48.33	*26*	43.33	*38*	63.33
5_4658	60	*0*	0.00	*18*	30.00	*21*	35.00	*25*	41.67	*26*	43.33	*45*	75.00
5_4648	61	*0*	0.00	*20*	32.79	*19*	31.15	*18*	29.51	*31*	50.82	*43*	70.49
5_4638	60	*0*	0.00	*25*	41.67	*7*	11.67	*22*	36.67	*18*	30.00	*42*	70.00
3_3771	60	*0*	0.00	*23*	38.33	*28*	46.67	*21*	35.00	*33*	55.00	*37*	61.67
3_3761	60	*0*	0.00	*19*	31.67	*25*	41.67	*34*	56.67	*34*	56.67	*37*	61.67
		**Damage State: ** ***Uvaesporites***											
		**Perfect**		**Thinned**		**Corroded**		**Broken**		**Pinched**		**Folded**	
**Sample**	***n*** ** Total**	***n***	**%**	***n***	**%**	***n***	**%**	***n***	**%**	***n***	**%**	***n***	**%**
5_4718	60	*1*	1.67	*36*	60.00	*54*	90.00	*25*	41.67	*26*	43.33	*15*	25.00
5_4678	36	*0*	0.00	*32*	88.89	*34*	94.44	*26*	72.22	*17*	47.22	*17*	47.22
5_4668	56	*1*	1.79	*36*	64.29	*44*	78.57	*18*	32.14	*18*	32.14	*27*	48.21
5_4658	60	*2*	3.33	*24*	40.00	*49*	81.67	*21*	35.00	*15*	25.00	*26*	43.33
5_4648	60	*1*	1.67	*29*	48.33	*49*	81.67	*21*	35.00	*24*	40.00	*26*	43.33
3_3771	60	*1*	1.67	*32*	53.33	*56*	93.33	*34*	56.67	*16*	26.67	*23*	38.33
3_3761	61	*0*	0.00	*49*	80.33	*58*	95.08	*37*	60.66	*20*	32.79	*24*	39.34

Data matrix of the frequency of each damage type in *Deltoidospora*, *Baculatisporites* and *Uvaesporites* from each of the samples investigated here. Details as for Table 2.

None of the plant beds at Astartekløft, including the Tr-J boundary interval in plant bed 5, are characterized by consistently high or low sporomorph damage frequency (Figure 3). There is a rise in the frequency of chemical damage frequency among random sporomorphs in plant bed 5, but the range of chemical damage frequency overlaps with the range recorded from other plant beds (Figure 3A). The frequency of chemical damage, particularly corrosion, is low among randomly selected sporomorphs from plant bed 6, but the range of chemical damage frequency overlaps with the range recorded from plant bed 5 (Figure 3A). The range of chemical damage frequency values among randomly selected sporomorphs from plant beds 6 and 7 do not overlap, and in both plant beds the maximum frequency of thinning is higher than the maximum frequency of corrosion (Figure 3A). The frequency of pinching is also noticeably higher than the frequency of folding and breakage in these two plant beds (Figure 3B).

### Comparison of Richness and Sporomorph Preservation Quality at Astartekløft

**Figure 4:**
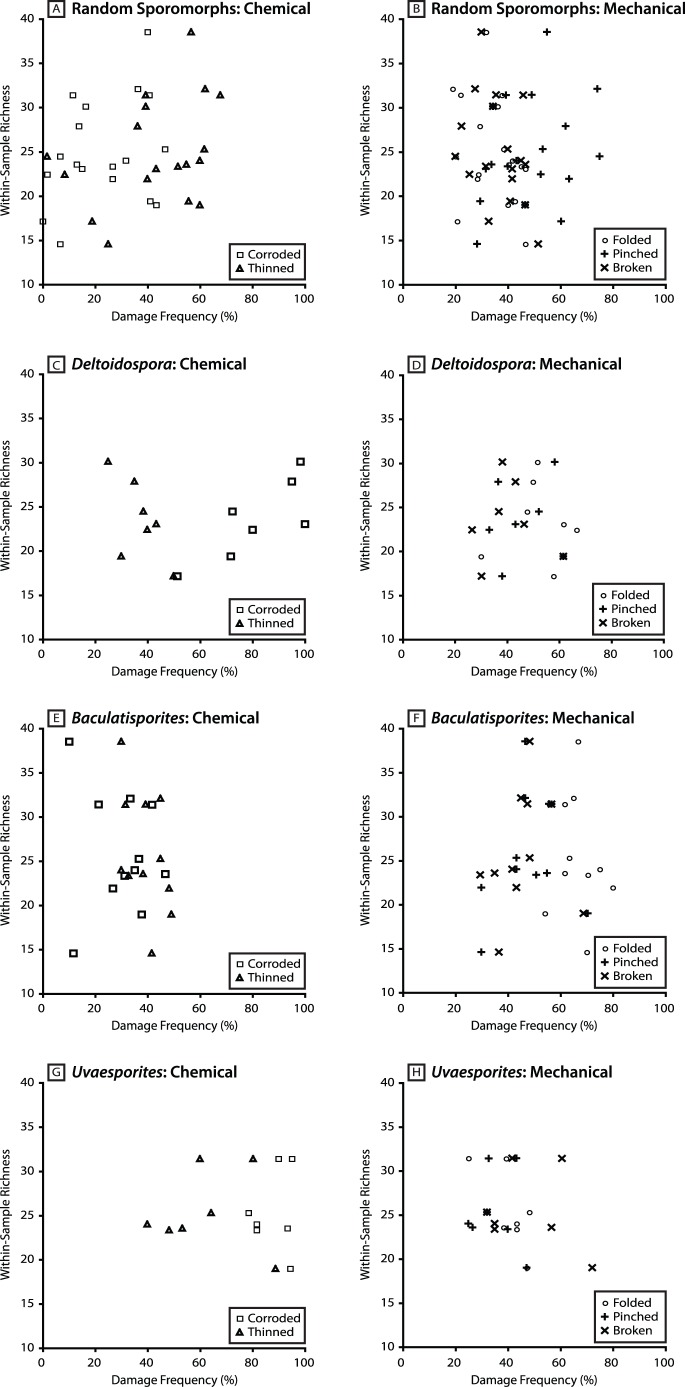
Scatter plots showing the relationship between the frequency of each damage type and within-sample sporomorph richness of each sample analysed in this study. Figures 5A, C, E and G show the relationship between the frequency of chemical damage (thinning and corrosion) and within-sample sporomorph richness. Figures 5B, D, F and H show the relationship between the frequency of mechanical damage (folding, pinching and breaking) and within-sample sporomorph richness. Perfect grains not shown. Within-sample richness from [13] (see also Table 1).

**Figure 5:**
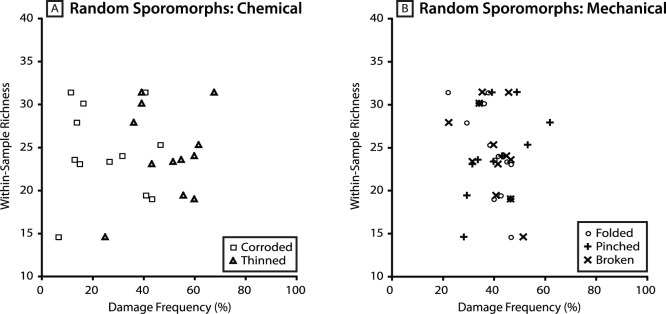
Scatter plots showing the relationship between sporomorph damage frequency among random sporomorphs and within-sample sporomorph richness for crevasse splay deposits at Astartekløft. Figure 6A shows the relationship between the frequency of chemical damage (thinning and corrosion) and within-sample sporomorph richness. Figures 6B shows the relationship between the frequency of mechanical damage (folding, pinching and breaking) and within-sample sporomorph richness. Perfect grains not shown. Within-sample richness from [13] (see also Table 1).

There is no evidence of a consistent correlation between the taxonomic richness of sporomorph assemblages and the frequency of damage among sporomorphs at Astartekløft. There is a negative relationship between taxonomic richness and the frequency of thinning in specimens of *Deltoidospora*, but there is a positive relationship between taxonomic richness and the frequency of corrosion in specimens of this genus (Figure 4C). Data points for all other groups of sporomorphs and damage types are widely scattered, so that samples with high richness may have high or low damage frequency, and samples with low richness may also have high or low damage frequency (Figure 4A, B, D–H). For example, the frequency of thinning among randomly selected sporomorphs in four samples that have low richness (5_4638, 14.57 taxa; 6_6106, 17.14 taxa; 5_4678, 18.97 taxa; 3_3715, 19.40 taxa; see Table 1) ranges from 19% (sample 6_6106) to 60% (sample 5_4678), and the frequency of pinching in the same group of samples and sporomorphs ranges from 28% (sample 5_4638) to 60% (sample 6_6106) (Table 2, Figure 4A, B). In comparison, the frequency of thinning among randomly selected sporomorphs in four samples that have high richness (3_3761, 31.38 taxa; 5_4718, 31.4 taxa; 7_7239, 32.09 taxa; 7_7269, 38.51 taxa; see Table 1) ranges from 39% (sample 3_3761) to 68% (sample 5_4718), and the frequency of pinching in the same group of samples and sporomorphs ranges from 39% (sample 3_3761) to 74% (sample 7_7239) (Table 2, Figure 4A, B). There is also no evidence of a consistent relationship between the taxonomic richness of sporomorph assemblages and the frequency of chemical and mechanical damage when only overbank (crevasse splay) deposits are considered (plant beds 3–5) (Figure 5).

## Discussion

### Causes of Sporomorph Damage at Astartekløft

The process of liberating sporomorphs from rock samples and storing them in slide preparations can result in damage to the exine [18]. All rock samples analyzed in this study were crushed prior to acid digestion, and this could result in mechanical damage to sporomorphs that is unrelated to the process of fossilization. For example, it seems likely that some of the sporomorphs scored as “broken” were damaged in this way by crushing. However, rock samples were crushed to cubes ∼5–10 mm^3^, and this would leave many sporomorphs isolated from the effects of crushing. Additionally, no samples from any particular stratigraphic horizon were crushed to a greater or lesser degree than others, and as a result the fraction of artificially broken sporomorphs is likely to be similar through the whole suite of samples investigated here, and at least among samples with similar sedimentological and lithological properties such as samples from plant beds 3–5.

**Figure 6:**
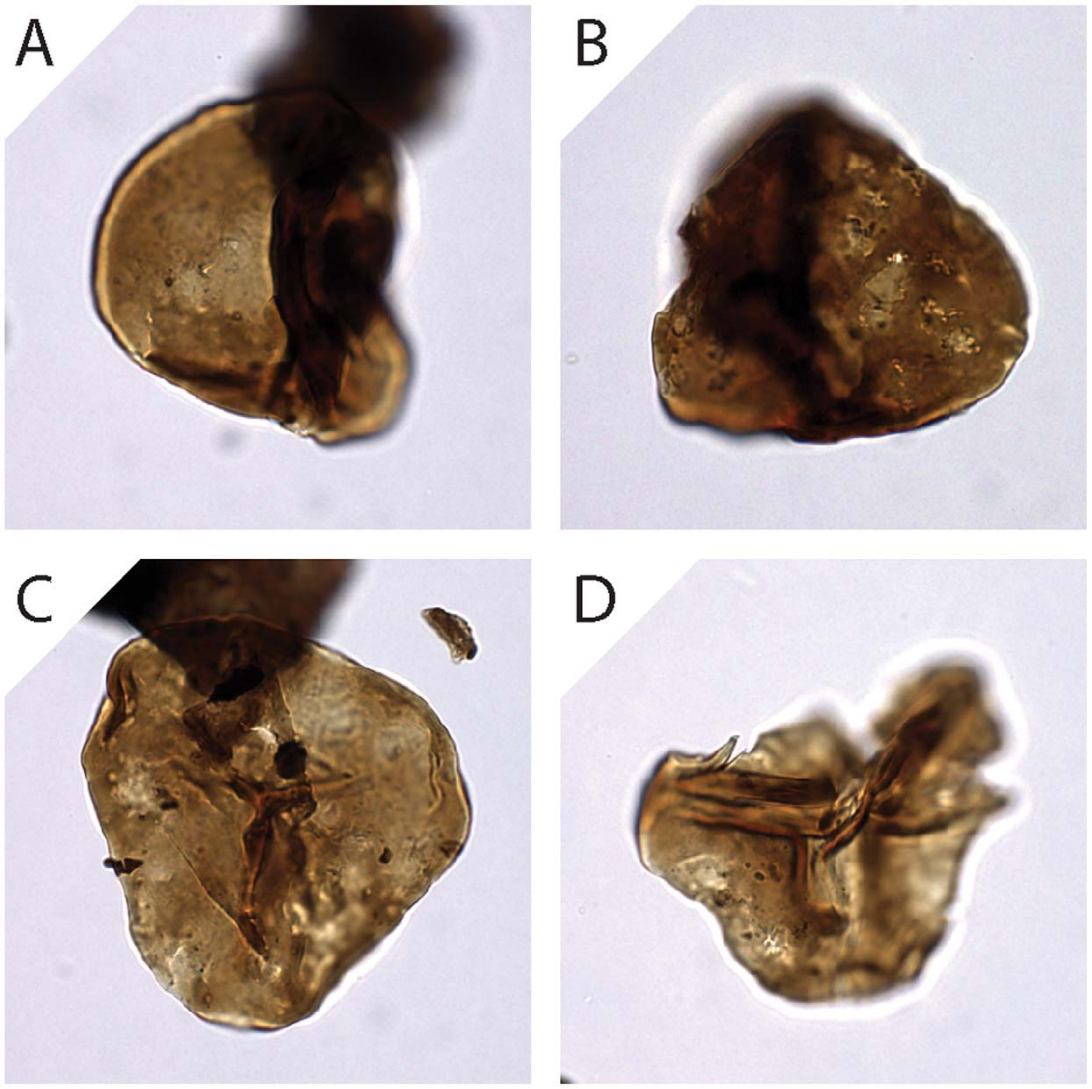
Spectrum of damage states in *Deltoidospora* from a single sample (4_4107). Note differences in corrosion intensity between 4A and 4B, and breakage between 4C and 4D.

Sporomorphs mounted in glycerine jelly can swell over time [27]. This effect can be significant, and sporomorphs mounted in glycerine are often considerably larger than sporomorphs mounted in other media such as silicone oil [28]. Such swelling can result in a thinning of the exine [27] that could be misclassified as “thinning” in our damage classification scheme (Figure 2). However, no pervasive glycerine swelling was observed in our samples and all sample residues were mounted in the same medium. Additionally, sporomorphs in a single sample record a range of damage states of varying intensity (Figure 6). It seems unlikely, therefore, that the effects of glycerine jelly have overprinted any trends in the frequency of sporomorph damage through time. Consequently, the patterns of sporomorph damage frequency reported here (e.g. Figure 3) are considered to be unrelated to the effects of sample processing and slide preparation.

**Figure 7:**
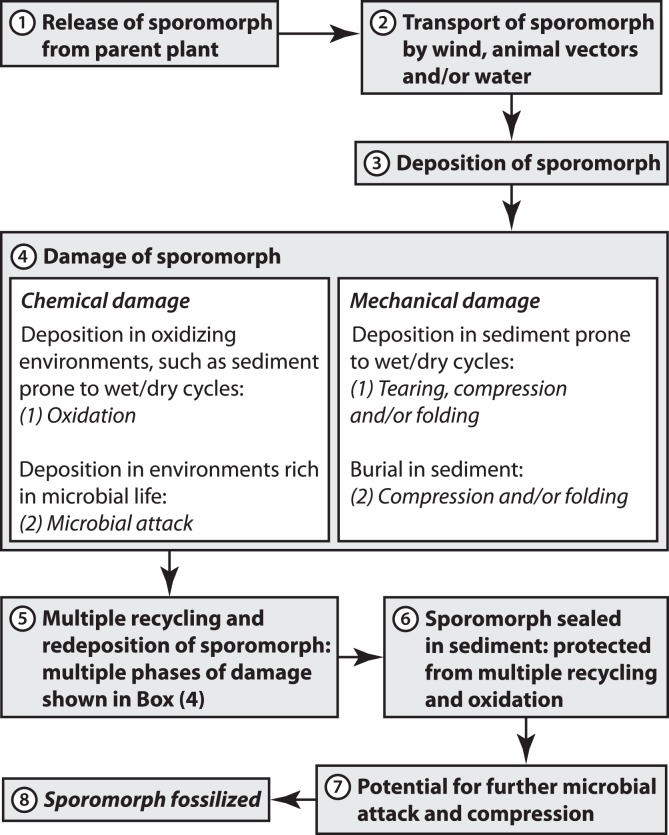
Flow diagram illustrating the types of damage that may occur to a sporomorph during the process of fossilization.

The types of damage that can occur during the fossilization of a sporomorph are illustrated in a flow diagram (Figure 7). In this diagram, emphasis is placed on the processes that occur after a sporomorph has been released from the parent plant and transported by wind, animal vectors and/or water. Chemical damage to the exine can occur if a sporomorph is deposited in oxidising environments, such as floodplain sediments that are prone to wet/dry cycles [19], and a sporomorph deposited in an environment that is rich in microbial life may suffer considerable microbial degradation [19] (Figure 7). Thinned sporomorphs (Figure 2) are interpreted here to reflect chemical damage, but it is possible that an immature sporomorph released early from the parent plant may have a thinner exine than a mature sporomorph from the same plant. Thermal maturity may also contribute to exine thinning. Future experimental work may help to understand the contribution of these three factors to the frequency of exine thinning in assemblages of dispersed sporomorphs. Mechanical damage such as breaking can result from the flexing of the exine during desiccation, which occurs when a sporomorph is exposed to wet/dry cycles in sediments [20], and also as a result of compression when a sporomorph is buried in sediment (Figure 7).

**Figure 8:**
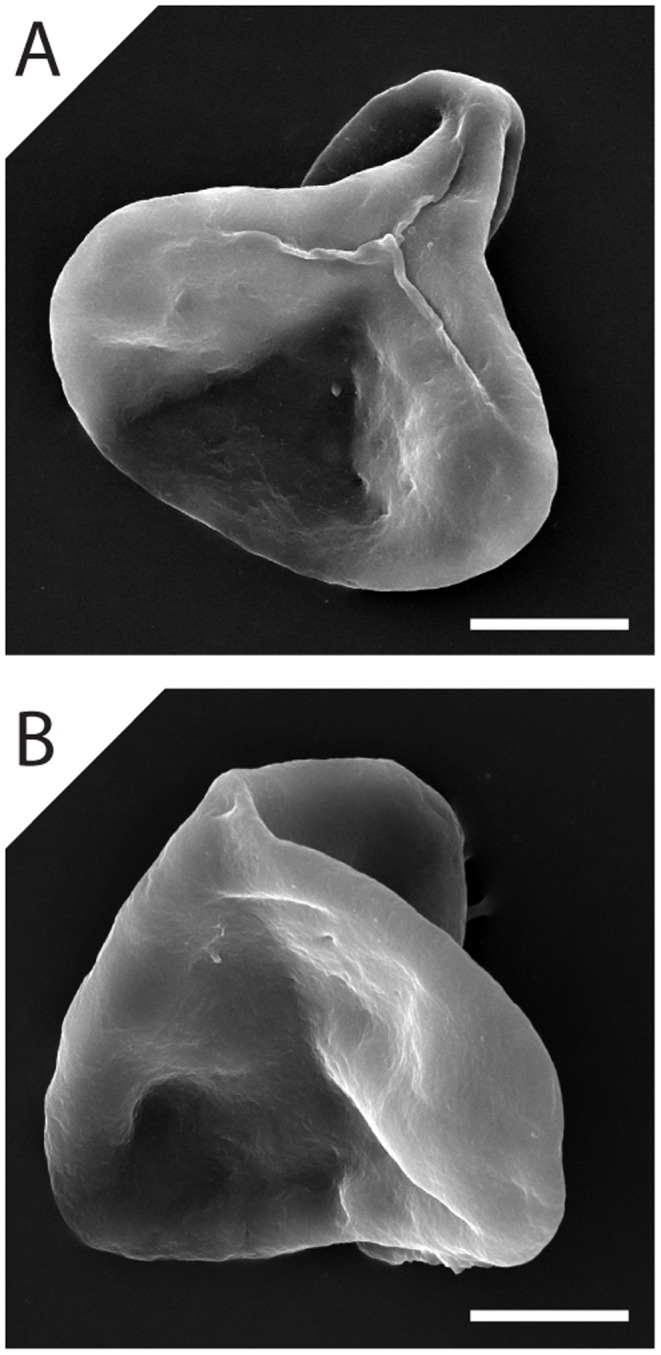
Scanning electron microscopy images of *Deltoidospora* showing two folding patterns that are noticeably common among specimens of this spore at Astartekløft. Note the thickened labrum of each laesura in Figure 8A. Figure 8B shows an example of ‘tri-plane folding’ [32], [33]. Scale bars represent 10 µm. Specimens of *Deltoidospora* were prepared for scanning electron microscopy (SEM) by picking individual spores from sample 6_6086 (Table 1) and mounting them onto a double-sided adhesive carbon disk attached to an SEM stub. The stub was then coated with gold-palladium using a sputter coater. The specimens were viewed using a JEOL JSM-6060LV scanning electron microscope at 15 kV.

It is thought that the patterns of chemical and mechanical sporomorph damage are strongly influenced by the primary morphology of a sporomorph. For example, the pattern of microbial attack follows the prominent rugulae on the surface of *Convolutispora*, whereas there is greater uniformity in the pattern of attack in the case of smooth or scabrate sporomorphs such as *Punctatisporites* [29]. Similarly, primary morphological features of a sporomorph exert a major control on the patterns of folding that are exhibited by a sporomorph. In the case of pollen grains, the number, arrangement, and shape of the apertures influence the folding pathway of a pollen grain during harmomegathic dehydration and hydration [30], [31], and sculptural elements such as the pila of lily pollen prevent mirror buckling of the exine during desiccation [31]. In the case of Tr-J sporomorphs at Astartekløft, some folding patterns are particularly common. Examples of two folding patterns that are noticeably common in specimens of *Deltoidospora* are shown in Figure 8. In the first common folding pattern, folding occurs in the interradial areas of the spore while the trilete mark remains undisturbed (Figure 8A). The thickened labrum of each laesura may increase the rigidity of the exine in this region of the spore [32], and this may encourage folding elsewhere. In the second common folding pattern, the spore has folded with its polar axis in the plane of compression (Figure 8B). This type of folding is known as ‘tri-plane folding’ [32], [33], and is also perhaps guided by the changes in exine rigidity created by the thickened labrum of each laesura.

### Variability in Sporomorph Preservation at Astartekløft

The variability in the frequency of sporomorph damage in samples from the same lithology and depositional environment at Astartekløft is a striking feature of our results (Figure 3). Such variability has been noted in studies of pollen preservation in the Quaternary [25], [26], and the frequency of corrosion among land pollen at sites in the northeast UK, for example, ranges from less than 5% to greater than 50% at Cess Dell and from 2% to 100% at Sproatley Bog [26].

**Figure 9:**
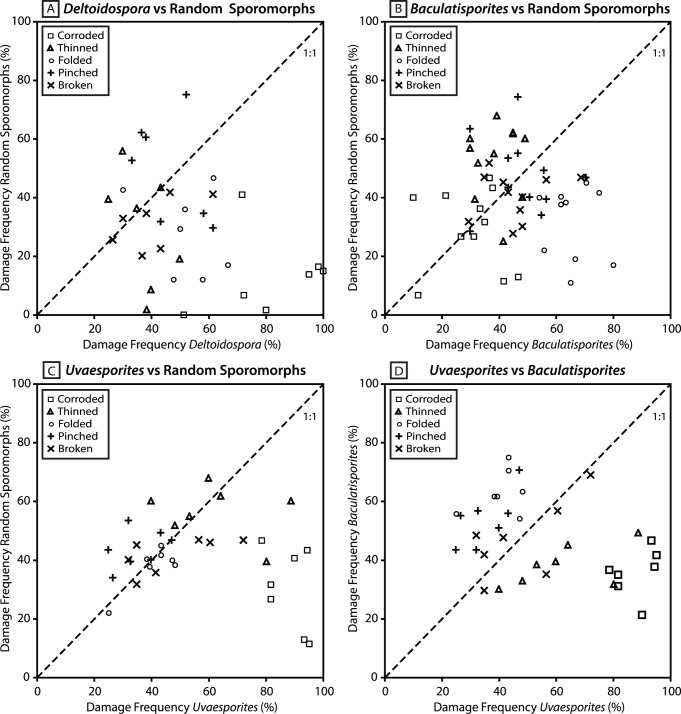
Scatter plots comparing the frequency of each damage type in random sporomorphs to the frequency of each damage type in *Deltoidospora* (8A), *Baculatisporites* (8B) and *Uvaesporites* (8C). Samples in which both random sporomorphs and a specific taxon were assessed are shown Table 1. Seven samples contained both *Baculatisporites* and *Uvaesporites* (Table 1) and the frequency of each damage type in these two taxa is compared in Figure 8D. Dashed diagonal line represents a line of equality.

At Astartekløft, this variability may partly be because different sporomorph taxa vary in their susceptibility to chemical and mechanical damage, reflecting differences in the thickness and chemical composition of the exine [34]. This is highlighted in Figure 9, which shows that there is no 1∶1 relationship between the frequency of each damage type in randomly selected sporomorphs and the frequency of each damage type in the three spore genera that have been investigated here. Instead, data points for most damage types are scattered across the line of equality that bisects each plot (Figure 9). Corrosion is consistently more prevalent in *Deltoidospora* and *Uvaesporites* than in randomly selected sporomorphs (Figs. 9A, C), and folding is consistently more frequent in *Baculatisporites* than in randomly selected sporomorphs (Figure 9B). Similarly, the frequency of each damage type in *Uvaesporites* is not matched by the frequency of each damage type in *Baculatisporites*. Chemical damage is more frequent in *Uvaesporites*, but folding and pinching are more frequent in *Baculatisporites* (Figure 9D), and this highlights that different sporomorph taxa can provide different views of the taphonomic conditions in a stratigraphic section. These observations also emphasize that tracking taphonomic regimes using single morphotypes is essential because variations in damage frequency through time may relate to changes in the composition of sporomorph assemblages rather than changes in taphonomic conditions.

However, preliminary data showing a spectrum of damage intensity in *Deltoidospora* from a single sample (Figure 6) also highlights variable sporomorph preservation that cannot be accounted for by differences in morphology between sporomorph taxa. Instead, such variability likely reflects the transport of sporomorphs from a mosaic of environments within the source area, each with their own taphonomic regime, and their subsequent mixing into a single sample.

### Sporomorph Preservation in the Late Triassic: a Signature of Tr-J Environmental Change?

It has been suggested that changes in the preservation state of sporomorphs might reflect environmental changes associated with CAMP volcanism at the Tr-J. In particular, it is thought that a pronounced darkening of sporomorphs in the Upper Rhaetian “Triletes Beds” of Germany, Denmark and Sweden, may have been caused by soil acidification from sulphuric acid deposition from CAMP volcanism [9]. Previous work has not highlighted a similar darkening of sporomorphs in sediments of Late Triassic age in East Greenland [13], [21], but the rise in chemical damage frequency among random sporomorphs in plant bed 5 is intriguing in this context (Figure 3A). Could the rise in chemical damage in plant bed 5 represent additional evidence for a widespread shift in taphonomic conditions for sporomorphs that is related to geological events around the Tr-J boundary?

The range of chemical damage frequency among random sporomorphs in plant bed 5 overlaps with the range of this damage type recorded in other plant beds (Figure 3A). In particular, the frequency of corrosion in one sample from plant bed 5 (sample 5_4638; 6.67%) is less than the frequency of corrosion in any samples from plant beds 3 and 4 (Figure 3; Table 2). Similarly, the frequency of corrosion in one sample from plant bed 3 (sample 3_3715) is higher than the frequency of corrosion in four samples from plant bed 5 (Figure 3; Table 2). A similar pattern of overlap can be seen in the frequency of thinning among random sporomorphs (Figure 3). Additionally, although there are only 2 data points in plant bed 3 and none in plant bed 4, both *Baculatisporites* (Figure 3E) and *Uvaesporites* (Figure 3G) record a fall in the frequency of chemical damage in plant bed 5, rather than a rise. Finally, the rise in chemical damage in plant bed 5 is likely related to processes such as chemical oxidation and microbial attack [19] rather than sulphuric acid deposition. This is supported by field observations of oxidation with iron staining on some rock samples from plant bed 5, although this did not seem to influence the preservation of leaf cuticle at this horizon. As a result, it seems problematic to connect the rise in the frequency of chemical damage among random sporomorphs in plant bed 5 to geological events at the Tr-J such as CAMP volcanism.

### Tr-J Vegetation Change and the Taphonomy of the Plant Beds at Astartekløft

The data presented in this paper build on previous taphonomic work at Astartekløft [11], and allow a clearer assessment of the degree to which the plant beds represent an equivalent suite of natural sampling conditions. There are no changes in depositional environment among plant beds 1–5 that are expressed at the outcrop scale [11], and there are no appreciable differences in the frequency of chemical and mechanical sporomorph damage among plant beds 3–5 (e.g. Figure 3). Although intriguing, it seems difficult to attach significance to the rise in chemical damage frequency among random sporomorphs in plant bed 5 because of the scatter and overlap of data points with samples from plant beds 3 and 4, and a fall in chemical damage frequency recorded by *Baculatisporites* and *Uvaesporites* (Figure 3). Taken together, these observations suggest that natural taphonomic regimes have not shifted radically between plant beds 1–5. This provides strong support for the view that vegetation change within these plant beds, such as the 10–12% decline in sporomorph diversity, peak plant extinction, and compositional change in plant bed 5, represents a real biological response to environmental change at the Tr-J [11]–[13].

Differences in plant diversity and vegetation composition between plant beds 1–5 and plant beds 6–7 should be interpreted with caution because of the differences in sporomorph and macrofossil source areas between the depositional environments represented by these plant beds [11], [13]. For example, fossils contained within the crevasse splay deposits (beds 1–5) may have been sourced from a large part of the catchment area of the hydrologically closed Kap Stewart Lake [35]–[38], whereas fossils contained within the poorly developed coal swamp (bed 6) likely represent a much smaller source area with a radius of ∼10–100 m [39], [40]. The frequency of chemical damage in plant bed 6 does appear lower than other plant beds, at least according to random sporomorphs (Figure 3A). However, despite major differences in source areas, the three depositional environments represented at Astartekløft do not appear radically different in terms of the processes responsible for the sporomorph damage states analyzed here (Figure 3).

In addition to characterizing natural taphonomic conditions at Astartekløft, the data presented here allow an assessment of the reliability of Tr-J sporomorph diversity records at this locality [13]. Specifically, our analyses do not provide any evidence of a consistent relationship between rarefied within-sample sporomorph richness and the frequency of damage among sporomorphs at Astartekløft, either when all environments of deposition are pooled (Figure 4), or when crevasse splay deposits are considered in isolation (Figure 5). This suggests that processes such as chemical oxidation, microbial attack and mechanical damage have not influenced the number of sporomorph taxa within samples from Astartekløft. Consequently, previously reported patterns of sporomorph richness across the Tr-J at Astartekløft are likely to be robust [13]. However, further work is needed to consider how reconstructions of plant diversity using pollen and spores might be affected by the selective removal of certain groups of sporomorphs by taphonomic destruction. This is vital because it has been shown that certain plant groups are under-represented in the sporomorph record compared to the macrofossil record at Astartekløft [13]. Specifically, preferential removal of dominant taxa in an assemblage with low evenness may lead to an increase in reconstructed diversity, whereas preferential removal of rare taxa may lead to lower reconstructed diversity. This should be noted when interpreting the relationship between within-sample richness and damage frequency among random sporomorphs at Astartekløft (Figure 4).

### Concluding Remarks

This study represents an example of how the frequency of sporomorph damage can be used as a guide to taphonomic regimes across an interval of global change. Investigations of this nature can help demonstrate that palaeobiological data spanning a presumed interval of biotic change in the geological past have been derived from an equivalent suite of natural sampling conditions [14]. This can provide confidence in palaeobiological data, which is crucial because such data may augment understanding of the current climate and biodiversity crises [41]. Our conclusions are as follows:

1. The frequency of each sporomorph damage type is extremely variable in samples from the plant beds at Astartekløft (Figures 3, 6 & 9). This variability likely reflects a combination of taxon-specific susceptibility to chemical and mechanical damage [34], and the mixing of sporomorphs from a mosaic of environments and taphonomic regimes into a single sample.
2. None of the plant beds at Astartekløft, including the Tr-J boundary interval in plant bed 5, are characterized by consistently high or low sporomorph damage frequency (Figure 3). There is a possible rise in the frequency of chemical damage frequency among random sporomorphs in plant bed 5, but this does not appear significant because of the considerable scatter and overlap of data points with samples from plant beds 3 and 4, and a fall in chemical damage frequency recorded by *Baculatisporites* and *Uvaesporites* (Figure 3). Consequently, this rise is not interpreted as evidence for a shift in taphonomic conditions for sporomorphs in East Greenland that is comparable to the pronounced darkening of sporomorphs reported in the Upper Rhaetian “Triletes Beds” of Germany, Denmark and Sweden [9].
3. Comparison of our results with previous work on the taphonomy of the plant beds at Astartekløft [11] confirms that natural taphonomic regimes did not shift radically between plant beds 1–5. This supports the view that vegetation change within these plant beds, such as the 10–12% decline in sporomorph diversity, peak plant extinction, and compositional change in plant bed 5, represents a real biological response to environmental change at the Tr-J [11]–[13].
4. We find no evidence of a consistent relationship between rarefied within-sample sporomorph richness and the frequency of damage among sporomorphs at Astartekløft (Figures 4 & 5). As a result, previously reported patterns of sporomorph richness across the Tr-J at Astartekløft [13] are likely to be robust.

## References

[1] Schoene B, Guex J, Bartolini A, Schaltegger U, Blackburn TJ (2010) Correlating then end-Triassic mass extinction and flood basalt volcanism at the 100 ka level. Geology 38: 387–390.

[2] Benton MJ (1995) Diversification and extinction in the history of life. Science 268: 52–58. 7701342 10.1126/science.7701342

[3] Deenen MHL, Ruhl M, Bonis NR, Krijgsman W, Kuerschner WM, et al (2010) A new chronology for the end-Triassic mass extinction. Earth and Planetary Science Letters 291: 113–125.

[4] Whiteside JH, Olsen PE, Eglinton T, Brookfield ME, Sambrotto RN (2010) Compound-specific carbon isotopes from Earth’s largest flood basalt eruptions directly linked to the end-Triassic mass extinction. Proceedings of the National Academy of Sciences, USA 107: 6721–6725. 10.1073/pnas.1001706107 PMC2872409 20308590

[5] McElwain JC, Beerling DJ, Woodward FI (1999) Fossil plants and global warming at the Triassic-Jurassic boundary. Science 285: 1386–1390. 10464094 10.1126/science.285.5432.1386

[6] Bonis NR, van Konijnenburg-van Cittert JHA, Kürschner WM (2011) Changing CO_2_ conditions during the end-Triassic inferred from stomatal frequency analysis on *Lepidopteris ottonis* (Goeppert) Schimper and *Ginkgoites taeniatus* (Braun) Harris. Palaeogeography, Palaeoclimatology, Palaeoecology 295: 146–161.

[7] Schaller MF, Wright JD, Kent DV (2011) Atmospheric *P* co _2_ perturbations associated with the Central Atlantic Magmatic Province. Science 331: 1404–1409. 21330490 10.1126/science.1199011

[8] Steinthorsdottir M, Jeram AJ, McElwain JC (2011) Extremely elevated CO_2_ concentrations at the Triassic/Jurassic boundary. Palaeogeography, Palaeoclimatology, Palaeoecology 308: 418–432.

[9] van de Schootbrugge B, Quan TM, Lindström S, Püttmann W, Heunisch C, et al (2009) Floral changes across the Triassic/Jurassic boundary linked to flood basalt volcanism. Nature Geoscience 2: 589–594.

[10] Ruhl M, Bonis NR, Reichart G-J, Sinninghe Damsté JS, Kürschner WM (2011) Atmospheric carbon injection linked to end-Triassic mass extinction. Science 333: 430–434. 21778394 10.1126/science.1204255

[11] McElwain JC, Popa ME, Hesselbo SP, Haworth M, Surlyk F (2007) Macroecological responses of terrestrial vegetation to climatic and atmospheric change across the Triassic/Jurassic boundary in East Greenland. Paleobiology 33: 547–573.

[12] McElwain JC, Wagner PJ, Hesselbo SP (2009) Fossil plant relative abundances indicate sudden loss of Late Triassic biodiversity in East Greenland. Science 324: 1554–1556. 19541995 10.1126/science.1171706

[13] Mander L, Kürschner WM, McElwain JC (2010) An explanation for conflicting records of Triassic–Jurassic plant diversity. Proceedings of the National Academy of Sciences, USA 107: 15351–15356. 10.1073/pnas.1004207107 PMC2932585 20713737

[14] Behrensmeyer AK, Kidwell SM, Gastaldo RA (2000) Taphonomy and paleobiology. Paleobiology 26: 103–147.

[15] Mander L, Twitchett RJ (2008) Quality of the Triassic–Jurassic bivalve fossil record in northwest Europe. Palaeontology 51: 1213–1223.

[16] Harris TM (1937) The fossil flora of Scoresby Sound East Greenland, Part 5. Stratigraphic relations of the plant beds. Meddelelser om Grønland 112(2): 1–112.

[17] Van Mourik JM (2001) Pollen and Spores, in Palaeobiology II (eds DEG Briggs, PR Crowther), Blackwell Science Ltd, Malden, MA, US.

[18] Traverse A (2007) Paleopalynology (2^nd^ edn.). The Netherlands: Springer. 813 p.

[19] Havinga AJ (1967) Palynology and pollen preservation. Review of Palaeobotany and Palynology 2: 81–98.

[20] Campbell ID, Campbell C (1994) Pollen preservation: experimental wet-dry cycles in saline and desalinated sediments. Palynology 18: 5–10.

[21] Pedersen KR, Lund JJ (1980) Palynology of the plant-bearing Rhaetian to Hettangian Kap Stewart Formation, Scoresby Sund, East Greenland. Review of Palaeobotany and Palynology 31: 1–69.

[22] Koppelhus EB (1996) Palynology of the lacustrine Kap Stewart Formation, Jameson Land, East Greenland. Danmark og Grønlands Geologiske Undersøgelse Rapport Appendix 5: 1–30.

[23] Cushing EJ (1967) Evidence for differential pollen preservation in Late Quaternary sediments in Minnesota. Review of Palaeobotany and Palynology 4: 87–101.

[24] Delcourt PA, Delcourt HR (1980) Pollen preservation and Quaternary environmental history in the southeastern United States. Palynology 4: 215–231.

[25] Jones J, Tinsley H, Brunning R (2007) Methodologies for assessment of the state of preservation of pollen and plant macrofossil remains in waterlogged deposits. Environmental Archaeology 12: 71–86.

[26] Tweddle JC, Edwards KJ (2010) Pollen preservation zones as an interpretive tool in Holocene palynology. Review of Palaeobotany and Palynology 161: 59–76.

[27] Sluyter A (1997) Analysis of maize (*Zea mays* subsp. *mays*) pollen: normalizing the effects of microscope-slide mouting media on diameter determinations. Palynology 21: 35–39.

[28] Holst I, Moreno EJ, Piperno DR (2007) Identification of teosinte, maize, and *Tripsacum* in Mesoamerica by using pollen, starch grains, and phytoliths. Proceedings of the National Academy of Sciences, USA 104: 17608–17613. 10.1073/pnas.0708736104 PMC2077075 17978176

[29] Moore LR (1963) Microbiological colonization and attack on some Carboniferous miospores. Palaeontology 6: 349–372.

[30] Wodehouse RP (1935) Pollen Grains. New York: McGraw Hill. 574 p.

[31] Katifori E, Alben S, Cerda E, Nelson DR, Dumais J (2010) Foldable structures and the natural design of pollen grains. Proceedings of the National Academy of Sciences, USA 107: 7635–7939. 10.1073/pnas.0911223107 PMC2867878 20404200

[32] Chaloner WG (1999) Plant and spore compression in sediments, in Fossil Plants and Spores: Modern Techniques (eds TP Jones, NP Rowe), Geological Society, London.

[33] Potonié R (1962) Regeln, nach denen sich die Sekundärfalten der Sporen bilden. Paläontologische Zeitschrift 36: 46–54.

[34] Havinga AJ (1984) A 20-year experimental investigation into the differential corrosion susceptibility of pollen and spores in various soil types. Pollen et Spores 26: 541–558.

[35] Muller J (1959) Palynology of recent Orinoco delta and shelf sediments. Micropalaeontology 5: 1–32.

[36] Gastaldo RA, Douglass DP, McCarroll SM (1987) Origin, characteristics, and provenance of plant macrodetritus in a Holocene crevasse splay, Mobile Delta, Alabama. Palaios 2: 229–240.

[37] Dam G, Surlyk F (1992) Forced regressions in a large wave- and storm-dominated anoxic lake, Kap Stewart Formation, East Greenland. Geology 20: 749–752.

[38] Hofmann C-C (2002) Pollen distribution in sub-Recent sedimentary environments of the Orinoco Delta (Venezuela) – an actuo-palaeobotanical study. Review of Palaeobotany and Palynology 119: 191–217.

[39] Jackson ST, Lyford ME (1999) Pollen dispersal models in Quaternary plant ecology: assumptions, parameters and prescriptions. The Botanical Review 65: 39–75.

[40] Harrington GJ (2008) Comparisons between Paleocene–Eocene Paratropical swamp and marginal marine pollen floras from Alabama and Mississippi, USA. Palaeontology 51: 611–622.

[41] McElwain JC, Punyasena SW (2007) Mass extinction events and the plant fossil record. Trends in Ecology and Evolution 22: 548–557. 17919771 10.1016/j.tree.2007.09.003

[42] Hesselbo SP, Robinson SA, Surlyk F, Piasecki S (2002) Terrestrial and marine extinction at the Triassic-Jurassic boundary synchronized with major carbon cycle perturbation: a link to initiation of massive volcanism? Geology 30: 251–254.

[43] Belcher CM, Mander L, Rein G, Jervis FX, Haworth M, et al (2010) Increased fire activity at the Triassic/Jurassic boundary in Greenland due to climate-driven floral change. Nature Geoscience 3: 426–429.

[44] Mander L (2011) Taxonomic resolution of the Triassic–Jurassic sporomorph record in East Greenland. Journal of Micropalaeontology. 30: 107–118.

[45] Kuerschner WM, Bonis NR, Krystyn L (2007) Carbon-isotope stratigraphy and palynostratigraphy of the Triassic–Jurassic transition in the Tiefengraben section – Northern Calcareous Alps (Austria). Palaeogeography, Palaeoclimatology, Palaeoecology 244: 257–280.

[46] Bonis NR, Kürschner WM, Krystyn L (2009) A detailed palynological study of the Triassic–Jurassic transition in key sections of the Eiberg Basin (Northern Calcareous Alps, Austria). Review of Palaeobotany and Palynology 156: 376–400.

